# Biomarkers in Rare Disorders: The Experience with Spinal Muscular Atrophy

**DOI:** 10.3390/ijms12010024

**Published:** 2010-12-24

**Authors:** Francesco D. Tiziano, Giovanni Neri, Christina Brahe

**Affiliations:** Institute of Medical Genetics, Catholic University of Sacro Cuore, Roma, Italy; E-Mails: gneri@rm.unicatt.it (G.N.); cbrahe@rm.unicatt.it (C.B.)

**Keywords:** spinal muscular atrophy, SMA, SMN, biomarker

## Abstract

Spinal muscular atrophy (SMA) is an autosomal recessive neuromuscular disorder caused by homozygous mutations of the SMN1 gene. Based on clinical severity, three forms of SMA are recognized (type I–III). All patients have at least one (usually 2–4) copies of a highly homologous gene (SMN2) which produces insufficient levels of functional SMN protein, due to alternative splicing of exon7. Recently, evidence has been provided that SMN2 expression can be enhanced by different strategies. The availability of potential candidates to treat SMA has raised a number of issues, including the availability of data on the natural history of the disease, the reliability and sensitivity of outcome measures, the duration of the studies, and the number and clinical homogeneity of participating patients. Equally critical is the availability of reliable biomarkers. So far, different tools have been proposed as biomarkers in SMA, classifiable into two groups: instrumental (the Compound Motor Action Potential, the Motor Unit Number Estimation, and the Dual-energy X-ray absorptiometry) and molecular (SMN gene products dosage, either transcripts or protein). However, none of the biomarkers available so far can be considered the gold standard. Preclinical studies on SMA animal models and double-blind, placebo-controlled studies are crucial to evaluate the appropriateness of biomarkers, on the basis of correlations with clinical outcome.

## 1. Introduction

Proximal spinal muscular atrophies (SMA) are a group of clinically variable motor neuron disorders characterized by the degeneration of the anterior horn cells of the spinal cord. On the basis of age of onset and of the maximum motor achievement, childhood-onset SMA is generally classified into three forms (type I to III). Type I is the most severe and common form of SMA and the most frequent cause of infantile mortality due to genetic cause. The onset of symptoms is generally between birth and six months of age, although in rare cases the first manifestations of the disease may occur during fetal life; patients show marked hypotonia, affecting mainly axial and proximal muscles, and do not achieve the seated position. Life expectancy is markedly reduced, generally less than two years of age, the main cause of death being respiratory insufficiency. Type II is an intermediate form, generally characterized by onset before 18 months of age. Affected children do not achieve autonomous ambulation. Type III is clinically the most variable form: the onset of symptoms is over 18 months of age, the motor milestones achievement is normal; patients may lose the walking ability at various ages. Type II and III patients generally experience long–term complications due to muscle weakness, inactivity and atrophy: 100% of type II and most type III patients present variable degrees of scoliosis, generally severe, and the majority have tendon retractions and joint contractures [1]. The classification into three forms does not fully reflect the clinical variability of these conditions, which is better depicted as a continuum, with many patients showing borderline phenotypes between two different forms. Indeed, alternative classifications have been proposed, including that of Dubowitz, who in 1995 suggested a decimalized classification for SMA patients which may more accurately describe the phenotypic complexity of the disease [2].

SMA type I-III are autosomal recessive conditions, caused by loss of function of the survival motor neuron (SMN1) gene [3]. Independent of the phenotypic severity, most patients (about 95%) have the homozygous deletion of the SMN1 gene, whereas about 2–3% of individuals are compound heterozygotes for the deletion of one allele and point mutations of the other (see Wirth for a review) [4]. SMN1 and a nearly identical copy, SMN2, are located in a duplicated inverted region at 5q13. Both genes encode the SMN protein, but due to alternative splicing, the majority of SMN2 transcripts lack exon7 (SMN-delta7), and are unable to produce a sufficient amount of protein to prevent the onset of the disease. The SMN protein is expressed ubiquitously and is localized in the cytoplasm and in the nucleus. It has been shown that the levels of SMN protein are markedly reduced in SMA patients, both in the spinal cord *in vivo* and in cell cultures, and inversely correlates with the phenotypic severity [5–7]. The SMN protein has different functions, including SNRNPs biogenesis and axonal transport, but it is not established yet which of the SMN functions is responsible for the pathogenesis of SMA [8]. Patients can carry variable copy number of the SMN2 gene, higher copy numbers being generally associated with milder phenotypes [9–11].

At present, no cure for SMA is available. One possible therapeutic approach is based on attempts at increasing the amount of SMN protein produced by SMN2 genes, through promoter activation, or reduction of exon7 alternative splicing, or both. Recently, evidence has been provided that SMN2 gene expression can be modulated *in vivo* and/or *in vitro*, using different strategies [12–28]. One possible alternative approach exploits the neuroprotective action of some compounds [29–31], aimed at preventing or delaying motor neuron loss. These compounds do not target the correction of the molecular defect of SMA since their mode of action is independent from the modulation of SMN2 gene expression. The efficacy of some compounds has been tested in clinical trials [21–23,32,33]. So far, none of the molecules evaluated has led to clinically meaningful improvements of motor function of patients.

The availability of various potential approaches for the treatment of SMA has raised a number of issues, including the choice of the most appropriate outcome measures, the duration of trials, the clinical characteristics of patients recruited, the study design (open label *versus* double-blind, placebo-controlled trials). These aspects have been reviewed by Kaufmann and Muntoni [34] and were the subject of a workshop which was held in London in 2008, with the participation of representatives of the European Medicines Agency (EMEA) and of TREAT-NMD, a network of excellence of researchers in the field of neuromuscular disorders, funded by the European Union. The final document of the workshop is available on the TREAT-NMD website (http://www.treat-nmd.eu/userfiles/file/general/EMEA_press_release.pdf).

Some issues are particularly relevant in the design of clinical trials for chronic disorders like SMA: the availability of data on the natural history of the disease, the reliability and sensitivity of outcome measures, the duration of the studies, and the number and clinical homogeneity of participating patients. Given the rarity of SMA, it is reasonable to anticipate that forthcoming double-blind clinical trials should involve patients and neuromuscular centers from different countries and should be internationally coordinated, in order to recruit a sufficient number of patients to gain clinically meaningful and statistically significant results.

**Scheme 1:**
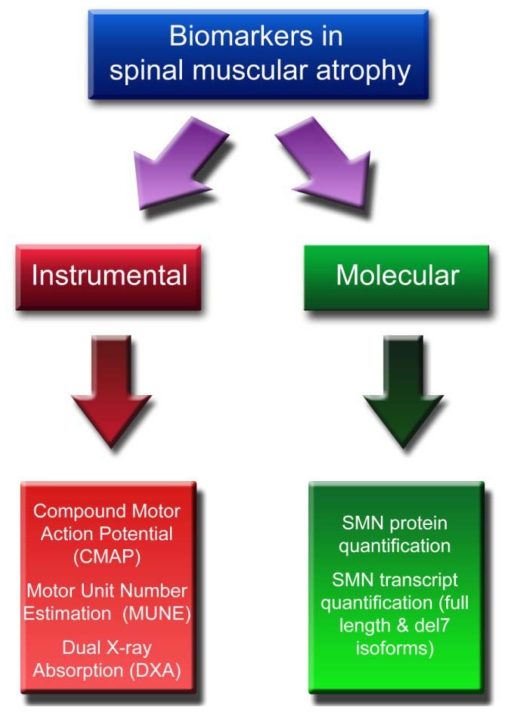
Schematization of biomarkers available in spinal muscular atrophy.

Equally critical is the availability of reliable biomarkers whose relevance in the field of SMA is related to different aspects. An objective measurement: (1) can overcome the risk of placebo effect, which has already been evidenced in our previous study of phenylbutyrate [22]; (2) allows comparison of clinically heterogeneous individuals, unlike most clinical-functional outcome measures; (3) can help to distinguish responder and non-responder individuals to a given treatment, as a wide variability in the response to some compounds has been reported [16,17], and (4) may shorten the duration of the trials. Two classes of putative biomarkers can be identified in SMA (see Scheme 1 and Table 1): instrumental and molecular. The Compound Motor Action Potential (CMAP), the Motor Unit Number Estimation (MUNE), and the Dual-energy X-ray absorptiometry (DXA) are included in the first group. Among molecular biomarkers, only dosage of SMN gene products, either transcripts (both full length and del7 isoforms) or protein, is currently available.

## 2. Instrumental Biomarkers

### 2.1. CMAP and MUNE

CMAP and MUNE are electrophysiological tools which allow the evaluation of skeletal muscle innervation status [35]. While CMAP size variations may not be specifically related to neurogenic conditions, MUNE helps to distinguish loss of motor neurons and reinnervations [35,36]. These two measures have been evaluated in patients affected with different neurodegenerative conditions, including SMA [37–39]. Available data suggest that these electrophysiological tools are potentially useful as biomarkers in SMA for several reasons. Swoboda *et al.* have performed a longitudinal natural history study on 89 SMA patients affected with forms of various severity and found that both measures had significantly lower values compared to controls and that they were related to the phenotypic severity [39]. However, while a clear difference was evident for different groups of patients, CMAP and MUNE were not predictive of the phenotypic severity of individual patients, since a certain degree of overlapping was observed among groups. Similar findings were reported by another study [37] that evaluated the strength of elbow flexion through a functional score (measured by the modified Medical Research Council scale) in 13 type-II/III patients. The data obtained, when related to MUNE values, indicated that the latter are not predictive of the functional outcome. However, in this study, the age range of patients and the number of years of disease elapsed from diagnosis was very wide, which may have impaired the functional evaluation of patients.

It has also been shown that CMAP and MUNE progressively decrease over time: Swoboda *et al.* found a progressive reduction of both parameters, more marked during the first years of postnatal life [39]. In type III patients, MUNE appeared to be more stable.

In our opinion, the results of electrophysiological evaluations should be integrated with the natural history data of the disease: in the case of SMA type I, it has been shown that the survival of patients has markedly increased over the last years, likely due to the more frequent application of proactive clinical interventions [40]; however, no data are available on the impact of longer survival on CMAP and MUNE variations. Regarding SMA type II, to our knowledge, some studies on the natural history were performed several years ago, without the confirmation of the molecular defect of the SMN1 gene [41,42] and, thus, these data should be interpreted cautiously. Deymeer *et al.* [43] performed the longitudinal evaluation of muscle strength of 10 type IIIb subjects, over a period of more than 10 years, and reported a slow progressive decline in muscle function of these patients, more evident in some muscle groups. The Authors related the functional decline to the progressive loss of motor neurons, rather than to the onset of complications of the disease, like joint contractures or scoliosis. The discrepancy between the results of Deymeer *et al.* and the stability of MUNE values reported by Swoboda *et al.* [39] in type III individuals, may be related to the different duration of the studies: A longer follow-up of patients through MUNE may disclose that, albeit slow, the loss of motor units is continuous. In our opinion, data on the natural history of the disease, both at clinical and instrumental levels, are critical for the identification of the endpoint and the duration assessment of clinical trials: while the stabilization or, hopefully, an increase in CMAP/MUNE could be considered as a marker of efficacy of a given compound in type I or type II patients, in the case of type III individual, this may be not sufficient.

It has been also shown that both CMAP and MUNE are related to SMN2 copy number [39]: Although the gene copy number is not predictive of the phenotypic severity of SMA of individual patients, the modifying effect of SMN2 genes has been demonstrated in several clinical studies, and also in murine models as SMA-like mice with higher hSMN2 copy number display milder phenotypes [44]. In our recent study on the effect of salbutamol treatment, we demonstrated for the first time that patients with higher SMN2 copy numbers have a better chance to respond to treatment at the molecular level (see below).

The use of CMAP and MUNE assessment in clinical trials as surrogate outcome measures has been evaluated in a recent open pilot trial of valproic acid in SMA patients [32], where Swoboda *et al.* found a statistically significant increase in CMAP (but not in MUNE) during treatment. However, it is not definitively established whether CMAP and MUNE are suitable as biomarkers and/or surrogate outcome measures in SMA since in addition to several pros, there are cons which undermine their applicability in clinical trials. In particular, there are still some crucial aspects that have not been defined: (1) CMAP has been evaluated in a double-blind study of valproic acid but the results of the study were not positive [33]; (2) CMAP and MUNE do not have prognostic value in individual patients, as discussed above, and (3) there is no evidence yet of possible correlations between variations of motor function and electrophysiological parameters.

### 2.2. DXA

DXA is the most generally accepted tool to measure bone mineral density (BMD) and it is based on X-ray absorption, generally evaluated at the level of lumbar vertebrae [45]. In most patients with limited motility, a reduction in BMD is often observed and it is a very common event in patients affected with neuromuscular disorders, like Duchenne muscular dystrophy (DMD) [46]. However, some studies have suggested that a primary bone remodeling defect may be present in SMA. In particular, Kathry *et al.* reported that young SMA patients show, as expected, a reduction of BMD compared to age-matched controls, but BMD reduction was significantly higher than that observed in age-matched DMD patients [46]. BMD loss was higher in non-ambulant SMA patients and in type II compared to type III. In another study, Kinali *et al*. found that in younger SMA patients (below 10 years of age) BMD was not reduced, but they did not observe the physiologic increase in BMD which normally occurs above this age, resulting in a relative reduction in BMD in SMA patients [47]. Interestingly, in a mouse model of SMA, Shanmugarajan *et al.* reported an osteoporotic phenotype in affected mice, suggesting a role of SMN protein in bone remodeling [48]. For these reasons, DXA is a candidate biomarker in SMA. To our knowledge, BMD has been evaluated only in one clinical trial of SMA patients, the open label of valproic acid cited above [32], where it increased during treatment compared to baseline. However, the biological and clinical significance of this finding is still unclear and it is not known whether it is related to the clinical outcome. Also in the case of this tool, double-blind, placebo-controlled studies are necessary to confirm the putative usefulness of DXA as a surrogate outcome measure in SMA clinical trials.

## 3. Molecular Biomarkers

At present, the dosage of SMN transcripts or protein in peripheral blood is the only potential molecular biomarker available. However, possible variations of SMN transcripts/protein levels as evaluated in leukocytes may not reflect the real effect of pharmacological treatment in target tissues, like the spinal cord and, possibly, skeletal muscle. Tissues other than blood, like skin or muscle biopsies, have been not considered so far for molecular biomarker analysis in SMA patients, due to the more invasive sampling procedures. This approach has been recently followed in a phase II clinical trial of the efficacy of PTC124 in patients affected by DMD who have undergone muscle biopsy before starting and after 24 weeks of treatment to evaluate the re-expression of dystrophin (see www.clinicaltrials.govwebsite). Further studies are necessary to evaluate the feasibility of a similar approach in SMA patients.

### 3.1. SMN Protein Quantification

SMN protein quantification is considered by most researchers as the most suitable and sensitive molecular biomarker for SMA. It has been shown that SMN protein levels are reduced in the spinal cord of SMA patients [4]. However, this is not demonstration for other tissues, like blood, which is the ideal target for biomarker analysis *in vivo*. Several techniques have been used for SMN protein quantification. Western blot was used in several *in vitro* and *in vivo* studies, mainly aimed at evaluating possible variations of SMN protein levels related to pharmacological treatment [17–18,24]. However, this assay has several limitations, essentially related to its semiquantitative nature, requiring normalization *versus* housekeeping proteins, whose levels are subjected to wide interindividual variations. An alternative approach has been proposed by Kolb *et al.*, who developed an immunoassay suitable for SMN protein quantification in peripheral blood mononuclear cells (PBMC), through which they could demonstrate a correlation with SMN2 copy number [49]. However, these authors found a reduction of SMN levels only in PBMC of type I patients, and did not find any correlation between protein levels and phenotypic severity. These findings clearly question the usefulness of quantifying SMN protein during clinical trials. ELISA assay is considered more sensitive and adequate for protein quantification since it does not require normalization to other proteins, given that SMN levels are quantified with respect to a standard curve constructed with serial dilutions of purified protein. To date, three different assays have been developed and validated. The first one, described by Thi Man *et al.*, is suitable only for *in vitro* applications [50]. More recently, Assay Design Inc. has developed a commercial assay, in collaboration with SMA Foundation, which has a very high sensitivity for SMN protein detection (Assay Designs^®^ SMN (human) Enzyme Immunometric Assay kit). The third assay was recently published by Piepers *et al.*, who used it to quantify SMN protein variations during valproic acid treatment of six type II/III patients [51]. These authors showed that their assay is sufficiently sensitive to measure SMN variations related to treatment, and also found that SMN protein levels in PBMC of patients are reduced compared to healthy controls. Although these results are promising, the small number of samples analyzed (only four healthy controls), the absence of age-matched controls, of a placebo arm and of clinical-molecular correlation, do not allow firm conclusions to be drawn on the validity of SMN protein dosage in clinical trials.

SMN protein dosage as a biomarker or surrogate outcome measure has some further technical drawbacks which impair the application of this assay in the context of multicenter double-blind clinical trials: PBMC should be processed within two hours from sampling to reduce possible biases due to cell death or to variations in protein levels; there are no commercially available stabilization buffers suitable to “snapshot” SMN expression at the moment of blood samplings; protein extraction requires larger peripheral blood draws often hard to obtain from very hypotonic or very young patients. Finally, SMN protein quantification is not indicated in the evaluation of those compounds whose mode of action is independent from SMN modulation, such as neuroprotective agents.

### 3.2. SMN Transcript Quantification

Several assays have been developed and validated for SMN transcript analysis, aimed at determining either full length (SMN-fl) or del7 (SMN-del7) isoforms, or the SMN-fl/del7 ratio [16,46–49]. These assays were developed for at least two purposes: (a) establishing whether SMN-fl transcripts are reduced in patients compared to controls, also in non-target tissues, like peripheral blood; and (b) evaluating the molecular effect of therapies aimed at modifying SMN levels *in vivo*. These therapies may be based on the activation of the SMN2 gene promoter, on the reduction of the alternative splicing of exon7, or both. To differentiate the effect of different therapeutic agents, it is essential to evaluate both SMN-fl and del7 isoforms, since promoter activation only would lead to an increase in both isoforms. On the other hand, if a therapeutic agent acts mainly by reducing the alternative splicing, an increase in SMN-fl levels and a concomitant reduction in the SMN-del7 isoform should be observed. Some researchers propose to evaluate possible variations of SMN-fl/del7 ratio only, but in this case, putative effects on promoter activation cannot be measured [18].

The results of different studies on SMN transcript levels have been discordant. In particular, a correlation between SMN2 copy number and transcript levels has been found by Sumner *et al.* [52] and by Vezain *et al.* [54], but not by Simard *et al.* [53] or by ourselves [55]. Vezain *et al.* and Tiziano *et al.* found a correlation between SMA phenotype and transcript levels, whereas Brichta *et al.* [17], Sumner *et al.* and Simard *et al.* did not. Significant differences between SMN-fl transcript levels of type I patients and controls were found by Brichta *et al.* and Sumner *et al.* but not by Simard *et al.* For the first time, we have recently shown that SMN-fl transcript levels are significantly lower in type II and III patients compared to controls, although not predictive of the phenotypic severity in individual patients [55]. The differences observed in different studies may be related to the methods used for SMN-fl level assessment. The majority of SMN mRNA assays are based on relative semiquantitative PCR in which transcript levels are determined by normalization with respect to housekeeping gene transcript levels, used as internal controls [52–54]. However, we and others have demonstrated that the expression levels of these genes vary widely in the general population and, or, can be probably affected by pharmacological treatments or metabolic status, thus reducing the sensitivity of the previously published assays [55,56]. The assay described by Brichta *et al.* is based on real time PCR and relative standard curves and SMN levels are measured as folds of variation compared to serial dilution of a control sample [17]. In our opinion, this approach may be considered unbiased only if the same sample is always used as control, otherwise it should be assumed that SMN levels in different control individuals are similar. We recently demonstrated that SMN-fl levels vary widely in control individuals, although they do not show a Gaussian distribution [55]. Our assay is based on absolute real time PCR. SMN-fl transcript levels are extrapolated from standard curves, constructed by serial dilutions of an external standard and are measured as number of mRNA molecules/ng of total RNA. The main advantage of this approach is that it allows quantification of SMN-fl levels independently from housekeeping control transcripts. On the basis of our results, the International Coordination Committee, a U.S. based organization of SMA researchers and clinicians aimed at harmonizing the outcome measures in clinical trials, has indicated our assay as the most appropriate to be used for SMN transcript analysis during clinical studies in SMA. Very recently, we applied this assay to evaluate the effect of salbutamol, a candidate compound for the treatment of SMA, on SMN expression [25]. Twelve patients were included in this study, who took oral salbutamol for six months: in all patients we found an increase in SMN-fl levels in PBMC, and all individuals at six months reached the median transcript levels of controls. However, at present it is not possible to establish whether the restoration of SMN levels in blood is predictive of the increase in spinal cord, which may be critical for the recovery of SMA phenotype. For the first time, we found also that the molecular response to salbutamol was higher in individuals with a larger number of copies of SMN2 genes, suggesting that these individuals are better responders to the compound and that SMN2 copy number can be included as a randomization parameter during the design of double-blind, placebo-controlled trials. The efficacy of other compounds modulating SMN expression has been evaluated *in vivo* by means of different mRNA quantification assays. In our previous study on the effect of phenylbutyrate, we found considerable variations both among different subjects and among different blood samples from the same subject [16]. In another open-label trial with valproic acid, SMN2 mRNA levels were found elevated in seven patients and unchanged or decreased in 13 patients [17]. Similarly, in the most recent open label study of the effect of valproic acid, fluctuation of SMN mRNA levels throughout drug treatment was reported in patients showing increased, decreased or unaltered levels [32]. In our opinion, the variability observed in the molecular response to treatment may be related to the different assays used for transcript analysis, to the different molecular efficacy of the compounds, or to the individual response of each patient (responder *versus* non-responder individuals).

Some technical advantages support SMN transcript analysis as a biomarker/surrogate measure for SMA, compared to protein analysis, including the availability of several stabilization buffers which allow the samples to be preserved from RNA degradation and gene expression variations for up to five days, and the small amount of blood (down to 0.5 mL) necessary for the assay. These aspects are very relevant in the context of multicenter trials, especially when dealing with severely hypotonic patients.

SMN transcript analysis is not free from some drawbacks which need to be considered if this tool is to be accepted as standard biomarker in SMA. First, it should be established whether SMN protein and transcripts levels are related. This is a critical issue, since to be therapeutically effective, the increase in SMN-fl levels should result in a comparable increase in SMN protein. The presence of such correlation is not obvious, since the dynamics and the half-life of mRNAs and proteins are not necessarily comparable. However, in our opinion, this aspect is more relevant in studies aimed at defining the intracellular mechanisms leading to SMN increase, rather than in the context of a clinical trial. Indeed, independently from transcript/protein level correlations, transcript variations may be suitable to identify responders and non-responders to a given treatment or may be predictive of the clinical outcome. The latter is another critical issue: the demonstration of correlations between clinical and molecular response to a given treatment are still missing. These data can be provided only by double-blind, placebo-controlled studies, due to the placebo effect which is very common in open label studies. Other aspects should be clarified before stating that SMN transcript analysis is a biomarker for SMA: (1) like in many other conditions, PBMCs are not target cells in SMA and it should be demonstrated that SMN levels in blood reflect those found in target tissues, *i.e*., the spinal cord but also skeletal muscle (to our knowledge, a single study on animal models is currently available [57]); (2) like SMN protein dosage, transcript analysis is not indicated for the evaluation of potential therapeutic compounds that do not modify SMN levels.

## 4. Conclusions

The recent move of SMA research from basic to clinical has raised the necessity to develop reliable clinical and biological markers to monitor the response of SMA patients to therapeutic interventions. While validated clinical tools have been developed, and a general consensus has been reached on the most suitable and reliable outcome measures, none of the biomarkers described above can be considered the gold standard. Indeed, while each of them presents certain positive aspects, there are still several crucial issues (summarized in Table 1) which should be resolved before stating that a biomarker for SMA is available. In our opinion, the most promising biomarkers are MUNE and SMN transcript quantification, in terms of feasibility, costs and availability of preliminary data. Two complementary approaches may provide the proof of concept needed for biomarker validation: preclinical studies and double-blind, placebo-controlled studies. Preclinical studies on SMA animal models can provide information on some issues like correlations between transcript and protein levels and between target and non-target tissues, being the phenotype of murine models the most extensively characterized. Double-blind, placebo- controlled studies are crucial to evaluate the appropriateness of biomarkers, on the basis of correlations with the clinical outcome.

## Figures and Tables

**Table 1:** Potential biomarkers in spinal muscular atrophy.

Potential Biomarker	Pros	Cons
**Instrumental**		
*CMAP and MUNE*		
	• Both measures are related to phenotypic severity	• MUNE does not appear related to motor function in a group of type II patients
	• Progressively decrease over time (MUNE is more stable in type III)	• There is no evidence yet of correlations between motor function and CMAP variations
	• Are related to SMN2 copy number	
	• Have been evaluated in an open phase II trial of valproic acid	
	• CMAP, but not MUNE, increases with VPA	
*DXA*		
	• Bone density increased after VPA treatment	• The biological significance of BMD reduction in SMA patients is not established
		• It is not known whether BMD variations are related to the clinical outcome of treatment
**Molecular**		
*SMN protein quantification*		
	• SMN protein levels, as determined by cell immunoassay, are related to SMN2 copy number	• SMN protein levels are not related to clinical severity
	• For cell immunoassay, small amount of PBMC are sufficient for SMN quantification	• No stabilization buffers are commercially available for total proteins
	• ELISA assay is sensitive down to magnitude of pg/mL of SMN protein	• PBMC should be manipulated within 2 hours from sampling
		• The minimum amount of peripheral blood necessary for SMN quantification is not known
		• It is not indicated for evaluation of candidate compounds which do not modify SMN levels
*SMN transcript quantification*		
	• Small amounts of blood (2.5 mL or less) are sufficient for mRNA quantification	• It is not known if protein and transcript levels are related
	• Several stabilization buffers are available for multicenter clinical trials	• It is unknown if transcript level variations are related to the clinical outcome of treatment
	• SMN transcripts are stable over time	• It is unknown if transcript levels in blood and target tissues are related
		• It is not indicated for the evaluation of candidate compounds which do not modify SMN levels
